# Next Generation Sequencing Discoveries of the Nitrate-Responsive Oral Microbiome and Its Effect on Vascular Responses

**DOI:** 10.3390/jcm8081110

**Published:** 2019-07-26

**Authors:** Melissa M. Grant, Daniel Jönsson

**Affiliations:** 1 School of Dentistry, Institute of Clinical Sciences, University of Birmingham and Birmingham Community Healthcare Foundation Trust, Birmingham B5 7EG, UK; 2 Swedish Dental Service of Skåne, 222 37 Lund, Sweden; 3 Department of Periodontology, Faculty of Odontology, Malmö University, 214 21 Malmö, Sweden

**Keywords:** oral microbiome, saliva, nitric oxide, nitrate, nitrite

## Abstract

Cardiovascular disease is a worldwide human condition which has multiple underlying contributing factors: one of these is long-term increased blood pressure—hypertension. Nitric oxide (NO) is a small nitrogenous radical species that has a number of physiological functions including vasodilation. It can be produced enzymatically through host nitric oxide synthases and by an alternative nitrate–nitrite–NO pathway from ingested inorganic nitrate. It was discovered that this route relies on the ability of the oral microbiota to reduce nitrate to nitrite and NO. Next generation sequencing has been used over the past two decades to gain deeper insight into the microbes involved, their location and the effect of their removal from the oral cavity. This review article presents this research and comments briefly on future directions.

## 1. Introduction

Oral microbiota are the second most complex niche of the human microbiome [1]; they have been associated with several non-communicative diseases, including cancer [2], Alzheimer’s disease [3] and cardiovascular disease (CVD) [4,5]. The current review will focus on the association between the oral microbiota and the most well established mechanistic pathway through which the oral microbiota may modify CVD, namely via the nitric oxide (NO) synthesis pathway.

In Europe, 3.9 million people die yearly from CVD, costing about 210 billion Euros per year in healthcare costs, productivity losses and informal care [6]. Hypertension is an important subclinical parameter for cardiovascular disease—about 54% of all stroke and 47% of ischemic heart disease are attributed to hypertension [7]. Hypertension is defined by WHO as systolic blood pressure ≥140 mm Hg and diastolic pressure ≥90, however it was recently reported that the definition of hypertension needs to be re-visited as lowering systolic blood pressure not to under 140 mm Hg, but to 120 mm Hg causes less fatal and non-fatal cardiovascular events. This also suggests that hypertension may be more important in CVD than previously described [8].

There are several important mechanisms behind hypertension, but one key underpinning is the L-arginine/NO synthesis pathway. In addition to hypertension, the NO synthesis pathway is important in the pathobiology of CVD via inflammation and platelet activation and aggregation [9].

## 2. The L-Arginine/NO Synthesis Pathway

In 1998, Robert F. Furchgott, Louis J. Ignarro and Ferid Murad were awarded the Nobel Prize for detecting and describing L-arginine/NO synthesis in cardiovascular physiology and disease. Furchgott described the accidental finding that acethylcholine, bradykinin, histamine and 5-hydroxytryptamine relax isolated preparations of blood vessels, but only when the endothelium is intact. In rabbit thoracic aorta prepared as a transverse ring, acetylcholine stimulation with muscarinic agonists caused dilatation, but not in aorta prepared as a helical strip. Later, it was noted that gently rubbing the endothelium of the transverse ring preparations similarly caused loss of smooth muscle cell relaxation. The endothelium-derived factor relaxing the smooth muscle cells lining the arteries was named endothelium-derived relaxing factor (EDRF) [10,11].

Following the discovery of EDRF, there was a wave of research inhibiting EDRF. What these inhibitors all had in common was their redox potency, except hemoglobin which inactivated EDRF by binding to it. This led to the suggestion that EDRF might be a free radical [12,13], and eventually Ignarro et al. [14] suggested that EDRF may in fact be NO, which at that time was not known to be synthesized in humans.

Then the question was where NO originates from, and in macrophages it was shown that NO originates from L-arginine [15,16]. Later, the enzyme responsible for the conversion of L-arginine to NO was identified as NO synthase. In addition to macrophages, NO was discovered in the central nervous system [17]. Depending on the system, different isoforms of NO synthase were identified, namely eNOS in endothelial cells, nNOS in the nervous system, and iNOS in the inflammatory system.

The inhibition of L-arginine and knockdown of eNOS in animal models led to the paradigm shift that hypertension is not due to increased resistance, but due to decreased conductance in the vascular system. The eNOS knockout mice also had increased endothelial-leucocyte interaction, platelet aggregation and thrombosis [18]. When knocking down eNOS in hypercholesterolemic ApoE mice, the eNOS-deficient mice had accelerated atherosclerosis [19]. Taken together, this work showed that the importance of eNOS and the L-arginine/NO synthesis pathway goes far beyond hypertension. In fact, NO from the eNOS activity of endothelial cells not only causes less contractility of the smooth muscle cells, but also results in inhibition of activation, adhesion and aggregation of platelets, less adhesivity of leucocytes and enhanced oxygen delivery from erythrocytes [20].

In 1986, Ludmer and coworkers [21] published a now classical study showing that in subjects with known CVD, acetylcholine resulted in a paradoxical NO-mediated vasoconstriction, in contrast to vasodilation in healthy subjects. Importantly, subjects with minimal CVD (angiographically) also displayed vasoconstriction. All three groups dilated in response to nitroglycerine. What this study suggested was that dysfunctional endothelial NO production and availability might precede the formation of clinically significant atherosclerotic lesions—today known as endothelial dysfunction. The clinical evaluation of endothelial function is through flow-mediated dilatation (FMD), today known as a subclinical predictor of CVD events [22] which is impaired in known CVD high risk groups, such as smokers and subjects with hypocholesteremia [23].

Endothelial dysfunction affects the L-arginine/NO-synthesis in two important ways—on a cellular level and on a molecular level. Endothelial cells can undergo phenotypical changes from healthy to a pro-inflammatory cell type. Healthy conditions, such as laminar blood flow, cause upregulation of transcription factors, such as Kruppel-like factors (KLF) 2 and 4, whereas athero-promoting flow and pro-inflammatory content of the blood cause upregulation of NFkB, eliciting a pro-inflammatory cell phenotype [24,25]. KLF2 promotes anti-CVD mechanisms, such as anti-inflammation by inhibiting the NFkB pathway [26,27], and anti-thrombogenic by inducing eNOS and thrombomodulin and reducing plasminogen activator inhibitor (PAI-1) [28]. Interestingly, KLF2 can be induced pharmacologically, via statins that exert atheroprotective effects via KLF2 [29]. NFkB is a pro-inflammatory transcription factor causing recruitment and activation of leucocytes at the site and subsequent endothelial dysfunction [24,25]. On a molecular level, the inflammatory micro-environment at an atherosclerotic lesion causes a net excess of reactive oxygen species (ROS), including superoxide which inactivates NO by forming peroxynitrite which could result in DNA damage and protein modification, but also through uncoupling of eNOS [30].

## 3. Microbiome Contributions to NO Synthesis

**Figure 1:**
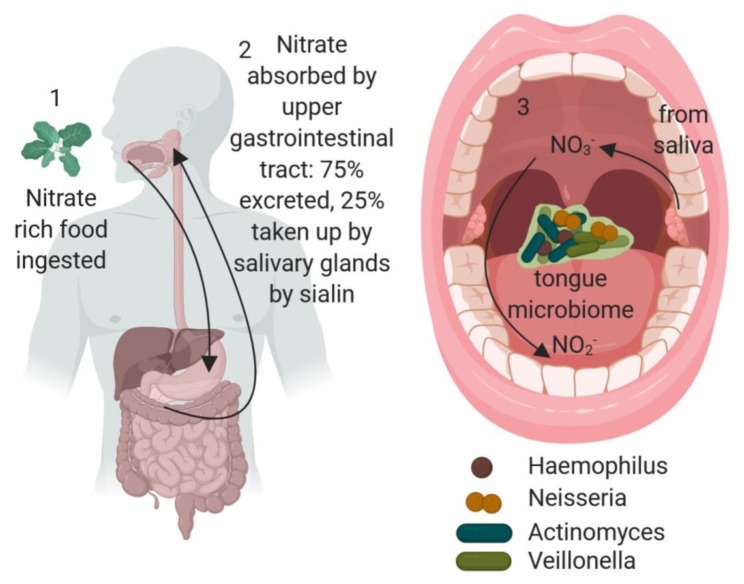
Enterosalivary nitrate production and the role of the oral microbiome. 1. Nitrate-rich food, such as leafy green vegetables, is ingested. 2. Nitrate is absorbed in the upper gastrointestinal tract and 25% is then found in saliva due to the action of the sialin anion transporter. 3. The oral microflora, particularly nitrate-reducing bacteria, such as *Actinomyces, Haemophilus, Neisseria* and *Veillonella*, residing in the dorsal tongue biofilm convert nitrate to nitrite, and also to nitric oxide (NO) which can be absorbed through the vascularized tongue or through swallowing back in to the gastrointestinal system for absorption. Image created in Biorender.

Research in the mid-1990s showed that NO production can be independent of NOS [31,32,33]. This production was linked directly to diet as, for example, nitrate-rich vegetable consumption could increase systemic nitrate and result in lowering of systolic blood pressure [34]. However, the activation of nitrate and transformation ultimately to NO requires its conversion to nitrite and mammals lack the enzymes required for this bioactivation. Termed the entero-salivary circulation, this requires consumption of nitrate which is absorbed by the upper gastrointestinal tract (Figure 1). Nitrate in circulation (approximately 25%, the remainder being secreted by the kidneys) is then selectively acquired by the sialin protein in the salivary glands and thus a high concentration of nitrate (1500 μM) is returned to the oral cavity [35]. There, the conversion of nitrate to nitrite is carried out by the oral microbiota [36]. Upon entering the stomach, the nitrite is protonated to nitrous acid (HNO_2_) which can then decompose to NO and other oxides. The following section reviews the results from next generation sequencing approaches to understanding more about the bacteria involved in the conversion of nitrate to nitrite in the oral cavity.

The first study examining the oral microbiota and the constituent microbes involved in nitrate and nitrite reduction was published in 2005 [36]. By mapping nitrate reduction across the mouth, Doel et al. could demonstrate that the majority of nitrate reductase activity was associated with the dorsum of the tongue. Indeed, the tongue dorsum has a distinct microbiome which is related to the other oral niches. It is more similar to the saliva microbiome than to the oral plaque microbiomes (Human microbiome project consortium 2012 [37]). They went further by isolating and culturing species associated within this site and verifying nitrate reductase activity. More species were found to have this activity under anaerobic conditions than aerobic conditions. Isolates of interest were 16S rRNA sequenced to gain their identity. The highest levels of nitrate reduction were found with *Actinomyces odontolytica* which was the second most common tongue isolate, though the authors also detected *Veillonella atypica, Veillonella dispar, Veillonella parvula, Actinomyces naeslundii, Actinomyces viscosus, Rothia dentocariosa, Rothia mucilaginosa, Staphylococcus epidermidis, Staphylococcus hemolyticus, Corynebacterium matruchotii, Corynebacterium durum, Haemophilus parainfluenzae, Haemophilus segnis, Propionibacterium acnes, Granulicatella adiacens, Selenomonas noxia, Capnocytophaga sputigena, Eikinella corrodens* and *Microbacterium oxydans*. The next report showing the microbiome associated with nitrate reduction was published a decade later, once the metagenomics analysis tools had matured more fully. Hyde et al. [4] used 16S rRNA and whole genome analysis of whole tongue scrapings and of biofilms originated from the scrapings matured on polymethacrylate (PMMA) discs for up to 4 days. The change in technique from Doel et al. [36] allowed for a larger number of operational taxonomic units (OTUs) to be discovered and a wider range of genera: *Streptococcus, Veillonella, Prevotella, Neisseria* and *Haemophilus*. The authors noted that there was a wide variation between different donors. With the samples that were used to inoculate biofilms, it was also noted that the number of OTUs dropped dramatically within the first 24 h and that by 4 days the biofilms were dominated by the *Streptococcal* species. Koopman et al. [38] also demonstrated this loss of diversity when growing saliva samples in a nitrate-reducing bacteria discovery study. This is of interest in light of the report by Doel et al. where culturing was used to find the nitrate-reducing bacteria before sequencing as this may have resulted in under-estimation of the discoveries. With the whole genome sequencing and pathway analysis, by Hyde et al., the pathways associated with the samples were more consistent with a match across the top eight pathways, such as various amino acid metabolic or synthetic pathways and nitrogen metabolism. Hyde et al. also split their results into samples with high, intermediate and low nitrate reductase activity: these samples were derived from the inoculated biofilms and were derived from individual donors creating a dataset of 30. Using principal coordinate analysis (PCoA), they showed that the samples with high nitrate reduction capacity were more likely to contain *Granulicatella, Veillonella, Neisseria, Actinomyces, Prevotella, Haemophilus, Fusobacterium* and some unclassified species of the *Gemellaceae* family. Some of these were already known whilst others were novel. Of note was the fact that *Lactobacillus* was associated with the least nitrate-reducing samples and the authors speculated that these genera may have an inhibitory role through production of some unknown byproduct that may inhibit nitrate reduction in those communities. Last, these authors also grew four microorganisms with putative nitrate and/or nitrite reduction capacity: *A. odontolyticus, V. dispar, F. nucleatum* and *S. mutans*. This last aspect of their study showed that these species could work independently or in a consortium to effectively remove nitrate and/or nitrite from growth medium. This is an important illustration of the complex interdependent networks in which biofilms exist. This paper not only confirmed previous findings but took them a step further, however both studies relied on discoveries from only 10 and 6 tongue scraping donors.

Hyde et al. [39] also published an article characterizing the tongue microbiome of rats, in comparison to human tongue scrapings, with a focus on the effect of dietary nitrate. One of the main findings was that the rat tongue microbiome was less diverse and appeared to be missing or had a greatly decreased amount of *Veillonella, Prevotella, Neisseria* and *Porphyromonas* in comparison to human samples. However, one of the limitations of the study was that different methodologies were used between the rat and human samples. Nevertheless, they could also examine the effect of administration of sodium nitrate via drinking water and chlorhexidine via oral rinse. The supplementation by sodium nitrate significantly decreased diastolic blood pressure and heart rate and made non-significant decreases in systolic blood pressure and mean arterial pressure. These changes were associated with relative increases in tongue *Haemophilus* species and *Streptococcus* species, which are nitrate and nitrite reducers, respectively. The change in the microbiome had not previously been demonstrated but later Koopman et al. [38] used a saliva inoculum from two human donors to create microcosms in the multiplaque artificial mouth (MAM) biofilm model system and to examine the effect of nitrate supplementation on the resultant microcosms. For one donor, *Neisseria* were associated with the nitrate-treated microcosms whereas *Veillonella* was more associated with nitrate treatment in the microcosms from the other donor. As both these genera seem to be missing from the rat tongue microbiome and this human study used saliva, it is difficult to draw a conclusion from a comparison of these experiments.

Chlorhexidine can abolish the effect of sodium nitrate supplementation [34,40,41,42] and Hyde et al. [39,43] showed that its use as a mouth rinse in rats decreased *Haemophilus, Aggregaterbacter,* and *Micrococcaceae* but increased *Enterobacteriaceae, Corynebacterium* and *Morganella* with the overall effect being an increase in diversity through a change in low abundance taxa in the baseline samples. Unfortunately, the chlorhexidine oral rinse did not change the blood pressure or heart rate measures as expected and the authors concluded that it did not remain in the mouth for long enough to exert the intended effect.

Recently, attention has turned towards responses in a wider range of donors. Vanhatalo et al. [43] examined the oral microbiome in young and old healthy donors and the response of these microbiomes to supplementation using a crossover design with either a high nitrate beetroot drink or placebo nitrate depleted over 10 days each. The supplementation of the high nitrate beetroot juice increased plasma nitrate to a similar extent whereas nitrite increases were greater for older participants. Mean arterial pressure, systolic blood pressure and diastolic blood pressure all showed a greater decrease with age and nitrite dose. Overall, they showed high quantities of *Fusobacterium nucleatum nucleatum, Prevotella melaninogenica, Campylobacter concisus, Leptotrichia buccalis, Veillonella parvula, Prevotella intermedia, Fusobacterium nucleatum vincentii* and *Neisseria meningitidis* in the tongue swabs. Changes in the oral microbiome were assessed in saliva samples before and after nitrate supplementation: supplementation changed the oral microbial communities but age did not and diversity was similar across the categories. Fifty-two taxonomic units were significantly changed with supplementation: *Veillonella* and *Prevotella* decreased, whereas *Neisseria* increased and there was no observed change in *Campylobacter* or *Haemophilus*. Burleigh et al. [44] studied the effect of nitrate-rich beetroot juice or nitrate-depleted placebo supplementation on the tongue microbiome and how the altered microbiome responded to an acute dose of nitrate. As seen before, there were decreases in *Prevotella, Streptococcus* and *Actinomyces* and an increase in *Neisseria* with supplementation. For the acute dose, saliva and plasma nitrate and nitrite were measured 1.5 h and 2.5 h, respectively, after nitrate consumption both before and after 7 days of supplementation. At both time points, there was an increase in nitrate and nitrite in both compartments but the alteration of the microbiome by 7 days of beetroot juice consumption did not alter the maximal nitrite and nitrate concentrations after the acute dose. The authors suggest that excess nitrite may be rapidly excreted to prevent excessive drops in blood pressure, or that the change in the nitrate/nitrite-reducing genera (*Prevotella* and *Actinomyces*) may balance the overall capacity of the system.

Kapil et al. [45] explored the influence of gender on nitrate reduction and the oral microbiota. Female participants had higher saliva, plasma and urine nitrite levels than males, and after supplementation with inorganic nitrate they showed a greater increase in plasma nitrate. However, there was no difference between the composition of the salivary microbiota of male and female participants. Ashworth et al. [46] considered the difference in the oral microbiome and dietary intake of inorganic nitrate between vegetarians and omnivores, as previously it was suggested that vegetarian diets are associated with lower blood pressure [47] and this is associated with higher nitrate intake [48]. By using dietary questionnaires, they demonstrated no difference in the consumption of nitrate and that saliva and plasma levels of nitrate and nitrite were similar between the two groups. The oral microbiome was similar between the two groups. The authors also administered a chlorhexidine mouth rinse intervention for 7 days and this caused a decrease in diversity in both groups and a drop in nitrate-reducing bacteria in both groups. Thus, this study suggested no differences in macrodiet and oral microbiome. This is in contrast to previous studies [49,50,51] which could be due to differences in the individuals examined across studies.

Tribble et al. [52] described the effect of tongue cleaning on the tongue microbiome and nitrate levels. Tongue cleaning is a technique that has been used for the removal of oral malodor or halitosis that is often ascribed to the tongue coating [53]. In this study, orally and systemically healthy oral health professionals were followed over the course of eleven days during which samples (tongue scrapings and saliva) were taken at baseline, at 7 days after use of the chlorhexidine mouth rinse and after a further 3 and 7 days after recovery from use of the mouth rinse. Chlorhexidine caused an increase in systolic blood pressure in the range of 5 mm/Hg which is equivalent to manipulation of dietary salt intake [54], however the donors were also stratified by the frequency of tongue cleaning as either none, once or twice per day. For those participants cleaning their tongue twice per day, there was the greatest increase in systolic blood pressure after the use of chlorhexidine. When examining the tongue microflora, the most common operational taxonomic unit was *Haemophilus parainfluenzae* and the second *Neisseria subflava*. However, the most common genus changed between individuals with groups of *Neisseria*, *Prevotella* and *Leptotrichia* as the most common. The frequency of tongue cleaning changed the abundance of species found: for example, *Leptotrichia spp* were detected in higher abundance on tongues that were not cleaned and daily cleaning (either once or twice) increased the abundance of *H. parainfluenzae* found. This showed that the tongue microbiome when tongue cleaning was implemented has a greater nitrate reductase capability. However, chlorhexidine treatment did not cause large-scale changes in the microbiome community. Further exploration suggested that any changes that occur are transient, with the recovery phase being associated with an increase in bacterial metabolic activity.

Box 1: Definitions:

- 16S rRNA is part of the bacterial ribosome, assigning structural scaffolding, and is of interest in the phylogenetic assignment of bacteria due to its slow rate of evolution.
- OTUs (operational taxonomic units) group together closely related individuals when individuals cannot be distinguished by the data available.
- Biofilms are embedded collections of microorganisms growing together in a matrix of extracellular polymeric substances on a surface.
- Next generation sequencing describes a wide range of DNA sequencing technologies capable of sequencing millions of fragments of DNA in parallel.
- Whole genome analysis sequences the whole of one or more genomes rather than a single gene, as with 16S rRNA analysis.
- Microbiome defines all of the microbial genomes in a given sample or environment.
- Microbiota defines the entire microbial flora in a given sample or environment.
- Principal coordinate analysis (PCoA) is a multivariate statistical technique used to reduce dimensions in data analysis and allowed for representation of the data visually.

## 4. Concluding Remarks

Overall, these next generation sequencing studies have demonstrated that there are nitrate and nitrite-reducing bacteria found in the mouth and that their removal through mouth rinsing with chlorhexidine will cause a temporary increase in blood pressure. The microbes most often found in the studies were from *Actinomyces, Haemophilus, Neisseria* and *Veillonella* genera. Samples have been from the tongue dorsum, where nitrate-reducing species were first identified, and also in saliva which may be easier to collect. The microbiomes of these two compartments are distinct but closely related. Furthermore, saliva indeed bathes the tongue, thereby enabling that microbiome to be sampled. More recent studies are just beginning to report [43,44,45,46,52] on how the microbiota in both the tongue dorsum and saliva change with different conditions, as previously only samples from homogeneous and healthy donors have been described. The number of donors in each study is also rising, helped by decreases in the costs of these types of experiments, which will help to gain more generalizable data and data that may reveal more subtle nuances between diseases types, ethnicities and other characteristics. This review has highlighted the role of the oral microbiota in the conversion of nitrate to nitrite and its importance to systemic balance. Understanding more about the role that the oral microbiota can play will enable future interventions that may aid with a stratified medicine approach that may rely more on bolstering the useful oral microflora and potentially reduce the use of antimicrobials.
